# Ultra-high-frequency Ultrasound to Assess Nerve Fascicles in Median Nerve Traumatic Neuroma

**DOI:** 10.7759/cureus.4871

**Published:** 2019-06-10

**Authors:** Antonio J Forte, Daniel Boczar, Jeremie D Oliver, Andrea Sisti, Steven R Clendenen

**Affiliations:** 1 Plastic Surgery, Robert D. and Patricia E. Kern Center for the Science of Health Care Delivery, Mayo Clinic, Jacksonville, USA; 2 Plastic Surgery, Mayo Clinic, Jacksonville, USA; 3 Plastic Surgery, Mayo Clinic, Rochester, USA; 4 Anesthesiology, Mayo Clinic, Jacksonville, USA

**Keywords:** ultra-high-frequency ultrasound, neuroma, nerve fascicles

## Abstract

A traumatic neuroma is a major cause of persistent neuropathic pain. Diagnostic imaging tools are critical to the success of surgical treatment. Ultra-high-frequency ultrasound is a novel technology that can generate frequencies up to 70 MHz, assessing structures up to 30 μm. We report a unique case of intraoperative use of ultra-high-frequency ultrasound to provide detailed imaging of nerve fascicles, facilitating surgical excision of the mass.

## Introduction

According to the National Institutes of Health, chronic pain is the leading cause of long-term disability in the United States. Traumatic neuroma stands as one of the most relevant causes of persistent neuropathic pain [1]. It is a consequence of a nerve-mending process which could affect any part of the body after external trauma or surgery. It is not classified as a neoplastic process but a disorganized proliferation of nerve axons, Schwann cells, and perineural fibroblasts following Wallerian nerve degeneration [2]. Ultra-high-frequency ultrasound (UHFUS) is a new technology in clinical diagnostic imaging. It can generate frequencies up to 70 MHz, assessing structures up to 30 μm. It is feasible to capture previously indistinguishable anatomic details, such as nerve fascicles [3]. UHFUS will soon become a widespread tool available at most hospitals, and its superior imaging of delicate structures should be tested on challenging lesions, such as traumatic neuromas.

## Case presentation

**Figure 1:**
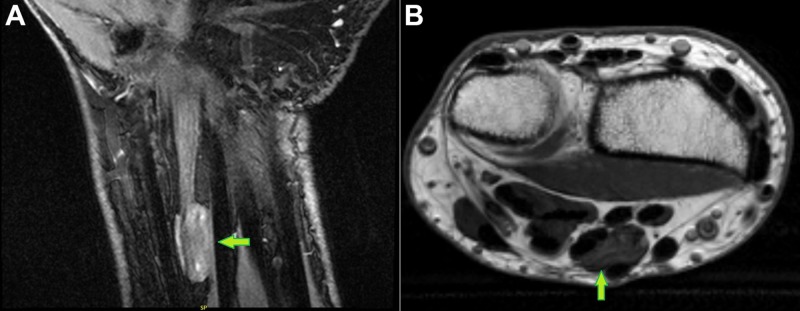
Magnetic Resonance Imaging of the Wrist Green arrows point to the median nerve mass: A) coronal view; B) axial view

A 71-year-old man presented with a history of right wrist pain for several years with increasing intensity over the past year and a half. Radiography showed advanced osteoarthritic changes. The patient also reported occasional paresthesias on the median nerve dermatome distribution. He stated that he had a wrist laceration 20 years prior with what he believed was a median nerve partial transection, leaving him with numbness in the thumb, index, and middle fingers that improved over time after the initial surgical nerve repair. As part of the pain and paresthesia evaluation, magnetic resonance imaging (MRI) was ordered. This study described a mass enlargement of the median nerve consistent with a peripheral nerve sheath tumor, such as a fibroma or schwannoma, with a heterogeneous increased T2 signal of the median nerve proximal to the carpal tunnel measuring approximately 16 mm x 10 mm x 6 mm (Figure 1). 

**Figure 2:**
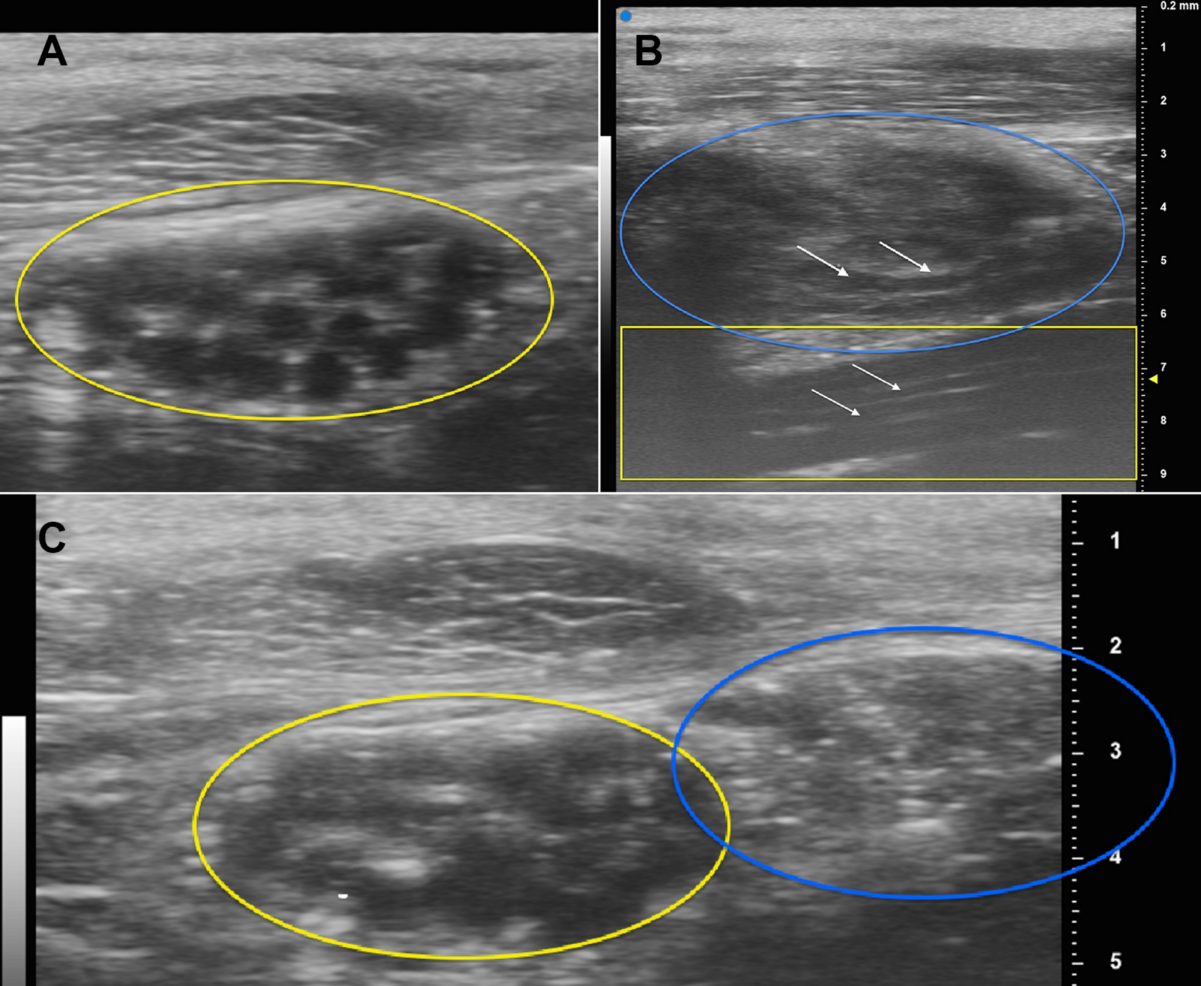
Ultra-high-frequency Ultrasound Images A) Cross-section of the median nerve distal to the neuroma (yellow oval); B) long axis of the neuroma (blue oval) and median nerve (yellow box); white arrows point to the long axis of the median nerve fascicles; C) cross-section of the median nerve (yellow oval) and neuroma (blue oval).

The nerve appeared normal proximal and distal to this level. The patient agreed to surgical exploration with UHFUS examination which helped delineate where the mass was in relation to the nerve fascicles (Figure 2). 

**Figure 3:**
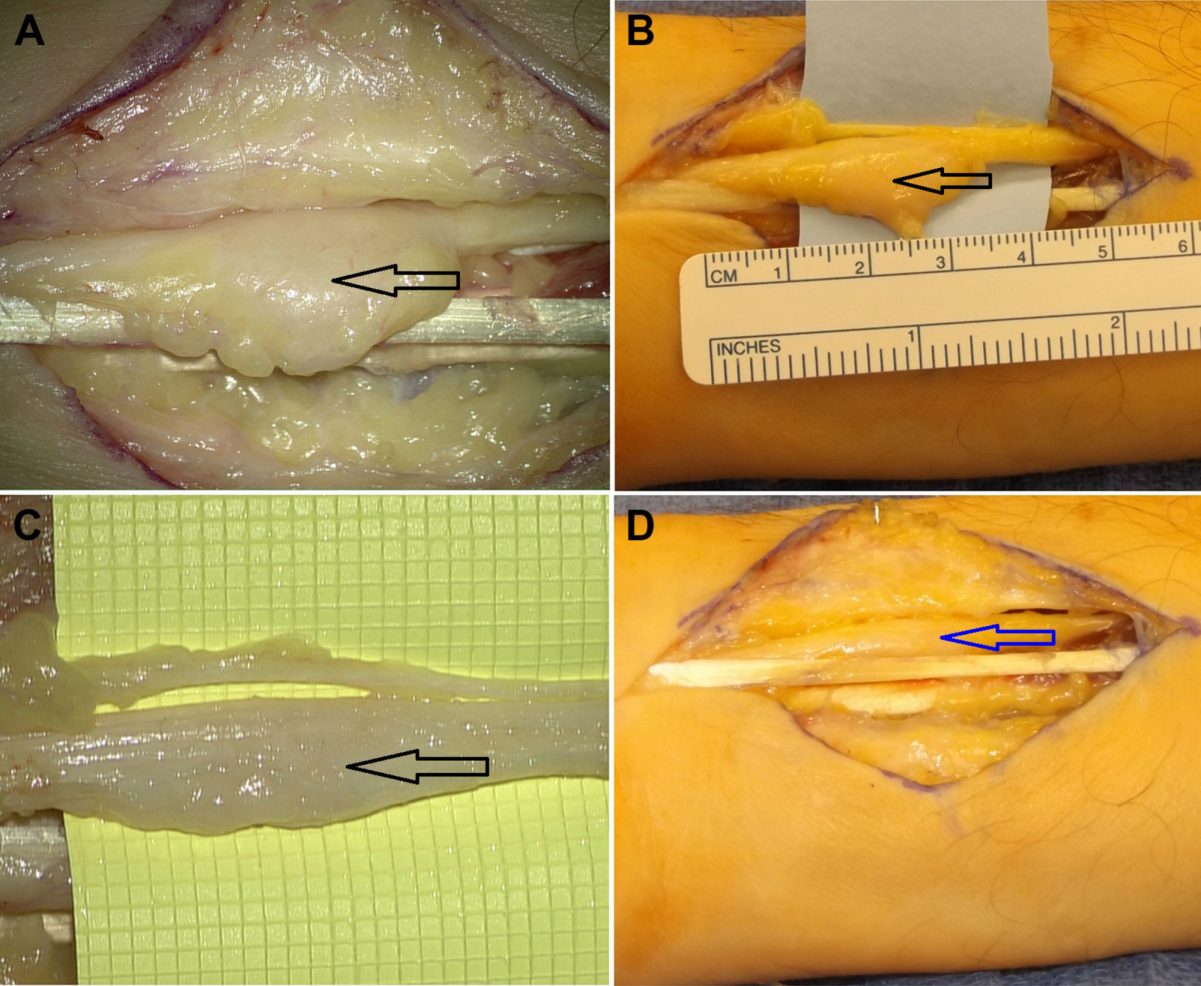
Intraoperative Image A-B) Before resection of the neuroma (black arrows); C) intraoperative microscopic view of the neuroma, X8 magnification (black arrow); D) macroscopic view after resection of the neuroma (blue arrow).

Under microscope magnification, an interfascicular dissection was performed and the mass was excised (Figure 3). 

Pathologic examination identified the mass as a traumatic neuroma.

## Discussion

UHFUS is a novel technology with few clinical applications described in the literature. Its capacity to visualize small structures can assess nerve fascicles, allowing surgical removal of neuromas with nerve function preservation. To our knowledge, this is the first report of the application of UHFUS in the evaluation of traumatic neuroma. Traumatic neuromas have anomalous anatomy, the consequence of adhesions, scar tissue, and foreign bodies [1]. Diagnostic imaging tools, such as MRI and ultrasound, are considered fundamental for the success of the operation, preventing postsurgical neurologic deficits [4-5]. Ulatowski and Kaniewska [6] provided some evidence that peripheral neural sheath tumors could be removed with nerve preservation. However, they suggested that these surgeries should be performed in microsurgery centers for careful intraoperative evaluation. MRI has been proven to be the most effective imaging modality for visualization of neuromas, but ultrasonography is frequently used due to lower cost and feasibility of dynamic images, allowing nerve continuity visualization [7-8]. Aggarwal et al. [9] pointed out that conventional ultrasound fails to determine nerve continuity in cases where fibrosis and architectural distortion are found, as in traumatic neuromas. Lee and Yoon [4] evaluated the ultrasonographic findings of a schwannoma of the hand and reported that diagnosis was challenging when using ultrasound alone because small lesions may mimic nerve ganglion, leading to a misdiagnosis. In our case, UHFUS examination assisted us in identifying this lesion as a neuroma instead of a schwannoma, as suggested by the MRI, allowing us to optimize fascicle identification and safely proceed with surgery. However, these higher ultrasonic frequencies have lower pulse lengths, which reduce tissue penetration and limit its utility to superficial anatomical structures.

## Conclusions

We report the use of UHFUS for detailed visualization of nerve fascicles previously inaccessible with other imaging modalities. A better awareness of the anatomy can guide surgeon decisions about nerve preservation, thus optimizing the procedure. We strongly encourage further studies addressing the use of UHFUS for neuromas and nerve tumors.
